# Multiple roles of DNA methylation in sea-ice bacterial communities and associated viruses

**DOI:** 10.1093/ismejo/wraf198

**Published:** 2025-08-30

**Authors:** Georges Kanaan, Jody W Deming

**Affiliations:** School of Oceanography & Astrobiology Program, University of Washington, 1503 NE Boat St, Seattle, WA 98195, United States; School of Oceanography & Astrobiology Program, University of Washington, 1503 NE Boat St, Seattle, WA 98195, United States

**Keywords:** Arctic, extremophile, epigenetic, metagenomic, regulation, memory, viral defence

## Abstract

Despite growing evidence for the role of DNA methylation in bacterial acclimation to environmental stress, this epigenetic mechanism remains unexplored in sea-ice microbial communities known to tolerate multiple stressors. This study presents a first analysis of DNA methylation patterns in bacterial communities and associated viruses across the vertical thickness of sea-ice. Using a novel stepped-sackhole method, we collected sea-ice brines from distinct horizons of an Arctic ice floe, capturing microbial communities that had been exposed to different environmental conditions. Through Oxford Nanopore sequencing, we characterized methylation patterns in bacterial and associated viral DNA, analysing for methylation motifs and differences between ice horizons. We identified 22 unique bacterial methylation motifs and 27 viral motifs across three nucleotide methylation types (5mC, 6 mA, and 4mC), with evidence of differential methylation between upper and lower ice. Analysis of metagenome-assembled genomes revealed the regulatory potential of methylation in both ice-adapted (*Psychromonas* and *Polaribacter*) and nonadapted bacteria (*Pelagibacter*); e.g. in *Pelagibacter*, differential methylation of the GANTC motif between upper and lower ice affected genes involved in core cellular processes. Viral methylation patterns showed evidence of recent infection. We also identified orphan methyltransferases in sea-ice phages, suggesting a mechanism for bypassing host restriction-modification systems and regulating host genes. Our findings reveal that DNA methylation serves functions in sea-ice beyond traditional restriction-modification systems that protect against foreign DNA, opening new avenues for research on the role of epigenetic mechanisms not only in acclimation to the cryosphere but also more generally in microbial ecology and evolution.

## Graphical Abstract

**Graphical Abstract:**
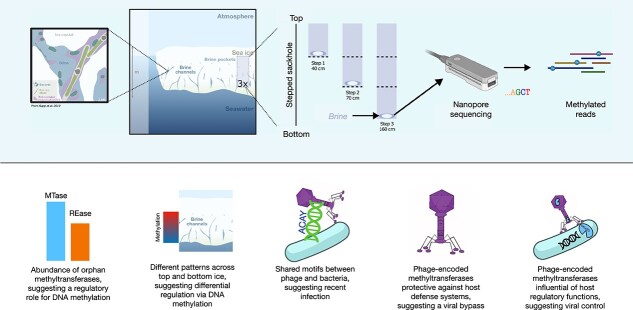

Permission from: Rapp Josephine Z, Sullivan Matthew B, Deming Jody W. Divergent Genomic Adaptations in the Microbiomes of Arctic Subzero Sea-Ice and Cryopeg Brines. *Front microbiol* 2021;12. https://doi.org/10.3389/fmicb.2021.701186ISSN=1664-302X

## Introduction

The dynamic environment of sea-ice represents one of Earth’s most extreme habitats, characterized seasonally by steep gradients of temperature, salinity, light, and space availability across relatively short distances [1]. Despite harsh conditions, sea-ice harbours diverse microbial communities, members of which are known to have evolved sophisticated adaptive mechanisms to survive and thrive within its challenging microenvironments [2]. Although numerous studies have examined the taxonomic composition, metabolic functions, and ecological interactions of sea-ice microbiomes [1, 3], the epigenetic mechanisms underlying microbial survival and acclimation in this extreme environment remain unexplored.

DNA methylation, the addition of methyl groups to specific nucleotide positions within the genome, represents one of the most fundamental epigenetic mechanisms across all domains of life [4, 5]. In prokaryotes, DNA methylation has been associated traditionally with restriction-modification systems that protect against foreign DNA [6]. Four types of restriction-modification systems exist, classified based on their structure and function. Type I systems are multisubunit complexes (R, M, and S proteins) requiring ATP to cleave DNA at variable distances from bipartite recognition sites. Type II systems employ restriction endonucleases and methyltransferases that act independently, cleaving at or near unmethylated recognition sequences. Type III systems combine R and M proteins into a heterodimer, methylating one DNA strand and cutting nearby, leading to incomplete digestion. Type IV systems lack methylase activity and exclusively target previously modified DNA (e.g. methylated or hydroxymethylated sequences) [7, 8]. Restriction-modification systems that have lost the R protein may leave behind an orphan methyltransferase, often from a Type II system. Orphan methyltransferases, common in bacteria [9] allow DNA methylation to play roles beyond defence, including regulation of gene expression, timing of DNA replication, and DNA repair [8, 10, 11]. These regulatory functions are particularly relevant in habitats where environmental conditions vary, requiring organisms to modulate their physiology in response [12, 13].

Sea-ice presents a promising natural system for studying these various roles of DNA methylation in the acclimation and adaptation of microbes to a dynamic environment. The vertical structure of sea-ice creates natural gradients in environmental conditions, with upper horizons experiencing greater fluctuations in temperature and brine salinity than less extreme and more stable bottom layers near the seawater interface [14]. The brines within sea-ice also host prokaryotic and viral communities in higher concentrations than in seawater, enabling investigation of methylation patterns across an environment where high cell-to-cell and cell-to-virus contact rates may accentuate interactions [15–17]. Sea-ice brines have long been hypothesized as sites for host-viral interactions due to the concentrating effects of the freezing process [18], which may give DNA methylation an outsized ecological role in this community.

The study of DNA methylation in environmental microbiomes has been revolutionized by recent advances in long-read sequencing technologies, particularly Oxford Nanopore sequencing, which can detect various forms of DNA modification directly during the sequencing process. This capability allows for characterization of methylation patterns at single-nucleotide resolution across entire genomes without the need for chemical treatment or enrichment steps and their associated biases. Despite these technological advances, our ecological understanding of DNA methylation at the community level, and how it may contribute to microbial acclimation to extreme conditions, remains limited. Previous studies have investigated methylation patterns in marine pelagic bacterial communities [19, 20], a freshwater bacterium [21, 22], a plant pathogen [23], model and pathogenic bacteria [24–27], and recently in a cultured psychrophile [28], but neither the methylome of a sea-ice community nor a community from any other extreme environment has been characterized, to our knowledge. This knowledge gap is notable given the unique environmental gradients and seasonal fluctuations presented by sea-ice and its importance as a model system for studying microbial adaptation to extreme conditions and analogous extraterrestrial environments [29, 30].

Here we present a multifaceted analysis of DNA methylation patterns across bacterial communities and associated viruses in sea-ice brines, the inhabited portion of sea-ice [27], focusing on methylation signatures in ice horizons across the environmental gradients inherent to sea-ice. Our objective was to collect brines from distinct horizons, using a stepped-sackhole method, to capture microbial communities with different environmental histories. All methods for sampling sea-ice microbes have limitations [31], but collecting brines directly *in situ* avoids biases due to osmotic shock and contaminants introduced by the traditional approach of melting ice into a melting solution. Using Oxford Nanopore sequencing and bioinformatic analyses, we aimed to characterize the methylation patterns of the recovered bacterial DNA, identify methylation motifs, and examine differences in methylation between ice horizons.

We hypothesized that bacterial methylation patterns would differ between upper and lower ice, as bacteria would use DNA methylation differently in response to more extreme and dynamic conditions (upper) versus more moderate and stable conditions (lower) in their distinctive horizons. Initially we focused on methylation as a regulatory mechanism in the bacterial communities and metagenome-assembled genomes (MAGs) obtained, documenting methylation motifs and differential methylation patterns between upper and lower ice. As we also obtained associated viral genomes, we followed with analyses of their methylated motifs and corresponding bacterial signatures, seeking evidence for host-viral interactions. We again found differential methylation across the ice and, unexpectedly, that methylated motifs in viruses may reveal infection histories. Additional roles for DNA methylation were suggested by the presence of phage-encoded methyltransferases. By elucidating the methylation landscape of sea-ice microbial communities and associated viruses, this study provides insights into epigenetic mechanisms underlying responses to an extreme environment and opens new avenues for research on the role of DNA methylation in microbial ecology and evolution.

## Materials and methods

Here we provide a brief description of materials and methods. Complete details for each section are available in Supplemental File S1.

### Ice floe drift

The ice floe’s drift path was reconstructed using sidrift commit #05075ca (Dr. Polona Itkin, https://github.com/loniitkina/sidrift). Environmental data (surface air temperature, sea-ice age/thickness, snow thickness, seawater salinity/temperature) were sourced from Copernicus Marine’s Arctic Ocean Physics Analysis and Forecast dataset cmems_mod_arc_phy_anfc_6km_detided_P1D-m [32, 33].

### Ice sampling

**Figure 1:**
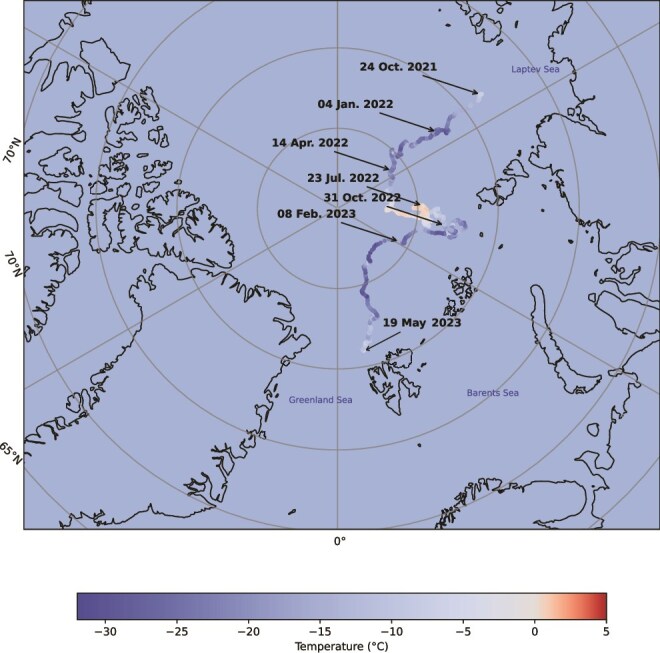
Atmospheric temperature along the floe drift path. Atmospheric temperature from October 2021 to May 2023, along the estimated drift path of the floe sampled in this study. Warm temperatures in summer 2022 and cold yet dynamic temperature variations throughout the ensuing year indicate that by sampling time in May 2023 the top of the sea-ice had experienced more extreme and greater variability in environmental conditions than bottom ice influenced by the relatively stable conditions of underlying seawater (Fig. S1).

Samples were collected during the BREATHE expedition (BR7008) on R/V *Kronprins Haakon*, drifting for 10 days over the Yermak Plateau, Arctic Ocean, 17–27 May 2023. Sackhole brines [34] were sampled at 81°2′55.94″N, 10°28′41.90″E on 21 May (Fig. 1 and S1). A stepped-sackhole method was used: holes were drilled to predetermined depths, brine was drained for at least 1.5 h per horizon, collected, and the process repeated until no further drainage. Depth horizons were defined by bulk salinity profiles: 0–40 cm (“top”), 40–70 cm (“middle”), and 70–160 cm (“bottom”). Four sackholes (1 m apart) were cored as environmental replicates (Table S1); due to sample loss, three were analysed. Brines were collected with a Masterflex peristaltic pump into acid-washed, Milli-Q rinsed Cubitainers. Adjacent ice cores sampled two days later, using a Kovacs Mark II system, were sectioned into corresponding horizons, and isohaline-melted to limit osmotic shock (Table S2) [31, 35].

### Shipboard processing

A blank control was filtered before samples. Brines were filtered at 1°C within 4 h onto 0.22 μm Sterivex® filters, flash-frozen in liquid nitrogen, and stored at −80°C. Brine salinity was measured on filtrate. Samples were labeled SX-Y by sackhole and horizon (Table S1).

### DNA extraction and sequencing

DNA was extracted using the Masterpure kit with modifications: Sterivex filters were cracked open, lysozyme digestion was added, and DNA pellet recovery used 14 000 × g centrifugation. DNA was quality-checked (NanoDrop), cleaned (Zymo MagBead Kit), and quantified (Qubit for later runs). Libraries were prepared with Oxford Nanopore kits and sequenced on a PromethION flow cell. Suboptimal yields were attributed to free adapter, polysaccharides, and low DNA input.

### Assembly, metagenomics, and bin curation

Reads <100 bp or Phred <Q10 were removed (Chopper [36]). Contaminant reads were filtered (SingleM [37], see File S2). Quality-controlled reads were co-assembled (Flye [38]), discarding contigs <1000 bp. Adapter/oligo contamination was removed (NCBI FCS tool [39]). Annotation and binning used Anvi’o, KEGG KoFams, COGs, pyrodigal, CONCOCT, METABAT2, and manual curation [40–47]. MAGs were assessed for redundancy/completeness (Anvi’o, CheckM2 [48]); coverage was determined with CoverM [49]. MAGs with >10% redundancy were discarded; only MAGs >30% complete were considered further. Viral contigs/plasmids were identified (geNomad [50], CheckV [51]). Taxonomy was assigned to bacterial MAGs using gtdb-tk [52], to contigs for community composition using Diamond [53] and MEGAN6-LR (database NCBI_nr 2025-01-05) [54], to contigs for methylation analysis using Kaiju (database refseq_nr 2024-08-13) [55].

### Methylation analysis

Reads were mapped (minimap2 [56]), and methylation was called per nucleotide (modkit, confidence ≥95%). *De novo* motif identification and differential methylation were performed with modkit. The nucleotide in a motif known to be the methylation site is bolded, e.g. [5m**C**]G. Only sites with sufficient coverage (≥5×) and statistical significance (modkit, *P* < .05) were considered. Genes for restriction-modification systems were identified using MicrobeMod [57]. Contigs were reordered for *Pelagibacter* MAG analysis of origin (*ori*) and terminus (*ter*) (Mauve [58], gc_skew [59]). Methylation fractions were normalized by coverage, with methylation levels categorized as low (<33%), medium (33%–66%), or high (>66%). Motif enrichment was tested (chi-squared), and differences between horizons were assessed (Kolmogorov–Smirnov with Benjamini/Hochberg correction, α = 0.01) [60].

### Software stack

Figures were generated with seaborn v0.13.2 [61], cartopy v0.23.0 [62], and matplotlib v3.8.4 [63]. Python v3.10.14 was used throughout. Data processing relied on polars v1.9.0 [64] and pandas v2.2.2 [65]. Virtual environments were managed with conda v24.1.2 and mamba v2.0.5. Workflows used snakemake v7.32.4 [66].

## Results and discussion

After providing the environmental and sampling context of the study, we describe sequencing and assembly outcomes, including limitations and quality controls. We then present a brief overview of the dominant members of the sampled communities revealed by metagenomic analyses, before focusing on DNA methylation patterns observed in bacterial and viral contigs. We highlight key findings regarding methylation motifs, restriction-modification systems, and evidence for regulatory and unexpected roles, supported by case studies of MAGs and prophages.

### Sampling and environmental history

The ice floe we sampled was 209 cm thick, with a snow cover of 2–11 cm across our sampling site and air temperatures that stayed close to −1°C, ranging between 0°C and − 5°C (Table S1). The salinity of sackhole brine samples confirmed them to be sea-ice brines with no seawater infiltration (see freeboard in Table S2). The floe was identified as first-year sea-ice, with a thin layer of second-year ice in some places (Figs. S2 and S3). To understand the environmental history of the sampled microbial communities, we mapped sea-ice related variables and atmospheric temperature along the floe’s drift path (Fig. S3). Atmospheric temperature drives temperature and salinity changes in the upper portions of sea-ice, while bottom ice remains relatively stable due to proximity to the ocean which remains at or above the freezing point. Mapping atmospheric temperature to the path travelled by the sampled ice floe since its formation (Fig. 1) confirmed that upper ice horizons experienced more variable and extreme conditions compared with the bottom ice as the temperature varied significantly over time. This contrast forms the basis of our hypothesis that different methylation profiles will be found across the ice as a response to different environmental conditions.

### Sequencing, assembly, and curation

To assess patterns in brine communities across the ice, we performed a co-assembly using long-read data from corresponding ice-core samples, which improved assembly accuracy (e.g. we were able to recover MAGs with low coverage in brine samples; Fig. S6) and yielded 101 882 contigs totalling 2.31 Gb. Read counts were generally lower than anticipated and varied between brine samples, but the control sample indicated little environmental or cross contamination during processing (Fig. S1). Sequencing difficulty was likely due to polysaccharide contamination in the extracted DNA, disrupting availability to the sensitive nanopores, and suboptimal DNA quantity. Future Nanopore sequencing efforts might benefit from additional DNA purification steps. Although useful for co-assembly, the ice-core data were excluded from analyses of methylated DNA as the melting procedure may artificially affect the community methylome.

Relative abundances should be interpreted cautiously given the uneven read counts between samples, despite being assessed conservatively for dominant organisms (Fig. S4). The MEGAN6 analysis, however, showed consensus in community composition across the sackhole brines, with ice-adapted genera *Polarella* and *Polaribacter* and seawater-adapted *Pelagibacter* abundantly present, as previously observed in Arctic sea-ice communities [1, 67–69].

We recovered eukaryotic, bacterial, archaeal, and viral contigs from the assembly, but focus here on bacterial and viral contigs. From 19 915 bacterial contigs we recovered 37 MAGs (File S2), six of which were >90% complete (Fig. S5), considered to be high-quality as defined by the MiXS standard [70]: *Lentimonas*, *Psychroflexus*, *Paraglaciecola*, *ASP10-02a* (Oceanospirillaceae), *Psychromonas*, and *Polaribacter*. Only the *Pelagibacter* MAG had sufficient coverage in both top and bottom samples (Fig. S6) for differential methylation analysis between horizons. For viral analyses, the 4896 contigs with a geNomad virus score above 90% yielded eight classified as complete, and 285 as high quality by CheckV.

### The bacterial methylation landscape

To assess the distributions of methylation motifs in bacterial contigs from sea-ice brines, we identified through homology analysis genes for restriction-modification enzymes recognizing 47 distinct methylation motifs. The most prevalent of these motifs were GANTC, GATC, and GGCC (File S3), with 6 mA as the most abundant nucleotide methylation type (63%). *De novo* identification yielded 22 unique methylation motifs across 1592 (8%) contigs; most (65%) had no detectable motif, whereas the remaining (27%) had insufficient data for identification. Most of these motifs were 5mC-based (97%), predominantly featuring [5m**C**]G. This methylation landscape thus shows striking differences between genetic potential and epigenomic observation: the encoded restriction modification enzymes mediate primarily 6 mA methylation, whereas *de novo* motifs were overwhelmingly 5mC-based. These proportions differ sharply from a pelagic metamethylome, where the most abundant methylation type was m6A (57%–63%), with only 15%–20% being 5mC [71], and 20 of the 22 motifs were not previously identified in a marine pelagic community [20]. Although perhaps unsurprising, given differences in community composition and sequencing approach and the conserved nature of selected motifs across taxa [71–75], this contrast suggests that sea-ice bacterial communities harbour methyltransferases and restriction enzymes with previously unidentified recognition motifs.

**Figure 2:**
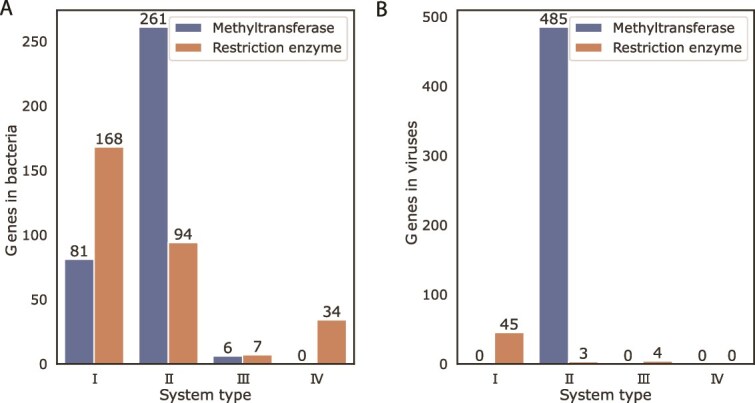
Number of genes involved in known restriction-modification systems in the sea-ice bacterial metagenome and the viruses. (A) Gene counts of the four known types of restriction-modification systems in the sea-ice bacterial metagenome. A surplus of methyltransferases (blue) over restriction enzymes (orange) suggests a high number of orphan methyltransferases, known to perform functions other than defence against foreign DNA, such as gene regulation and DNA repair. (B) Gene counts in the viruses associated with the sea-ice community. Only three types of restriction-modification systems were detected, but the surplus of type II methyltransferases (>10x the restriction enzymes) again suggests orphan methyltransferases with regulatory or DNA repair functions. The surplus holds when considering only *Caudoviricetes* (376 methyltransferases versus 48 restriction enzymes)*.* A complete list with E-value and homology information, quantifying the accuracy of these results, can be found in File S3.

To estimate the balance between defence and regulatory (or other) roles of DNA methylation in the sampled brine communities, we estimated the number of orphan methyltransferases in bacterial contigs. We found a surplus of orphan methyltransferases (Z-scores of 5.51 for Type I, and 8.86 for Type II, equivalent to *P* < .01) (Fig. 2). Their abundance suggests that many bacteria were using DNA methylation for regulatory roles rather than defence.

**Figure 3:**
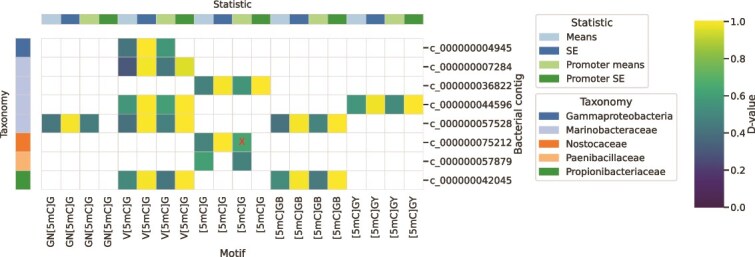
Significance of differential methylation in bacterial contigs between top and bottom ice. The d-value of the Kolmogorov-Smirnov similarity test is shown for each of the eight bacterial contigs with sufficient data. Red “X” signifies that the statistical test result is insignificant *(P* ≥ .05), and thus the methylation distribution for this contig can be considered the same between horizons. D-value of 1 indicates largest difference in distribution, 0 indicates no difference. Most contigs show significant differences in the distribution of per-nucleotide mean methylation fraction, when considering all motif instances and only those within promoter regions. Significant differences in standard error of the distributions suggest possible differences within the population.

To investigate DNA methylation as a mechanism for gene regulation under different environmental conditions, we compared methylation fractions between top and bottom ice of motifs occurring on the eight bacterial contigs with sufficient coverage. We found that all had statistically significant differences in methylation fractions between ice horizons (Fig. 3). Some showed differences in promoter-only regions between ice horizons, suggesting that gene expression was differentially regulated. Of the 182 instances of differentially methylated motifs across these contigs, none was contained in, or flanked by, genes with known functional annotation. Although we cannot indicate what function DNA methylation may have been regulating, these findings provide significant evidence for a regulatory role of DNA methylation in the bacterial community response to environmental differences across the ice. We can hypothesize that logical targets for this regulatory role would be genes related to acclimation to sea-ice conditions, such as extracellular polysaccharide production, osmolyte regulation, and extracellular enzyme production [76–79].

MAGs allow for understanding the genetic context of DNA methylation and interrogating its role in specific taxa. Of the 37 MAGs recovered from the sea-ice brines, we focused on the four with *de novo* methylation motifs (Table 1). *Psychromonas* and *Pelagibacter* are characterized later, as more information was available; *Polaribacter* and *Paraglaciecola* could be examined for defense versus regulatory roles.

**Table 1:** The metagenome-assembled genomes with identified methylation motifs and presence of orphan methyltransferases, which suggest DNA methylation function beyond immune defense.

Metagenome-assembled genome	*De novo* methylation motifs	Motifs of homologous enzymes	Number of orphan methyltransferases
s__paraglacieocola_sp913058035__bin_4_1_1-contigs	G**A**TC	GATC	2
g__pelagibacter__bin_0_9_3-contigs	G**A**NTC	G**A**NTC	1
g__psychromonas__bin.102	G**A**TC	GATC	1
g__polaribacter__bin_0_8_1-contigs	**C**CWGG, C**C**GG	GGCC, AAGACC	0

In the *Polaribacter* MAG, we found five restriction modification operons, each with a methyltransferase and restriction enzyme system of type I (1), type II (3), or type III (1). The motifs were recognized separately by methyltransferases in separate operons of type II and type III, GGCC, and AAGACC, respectively, though the latter had only 58% shared identity with the REBASE reference. Three putative orphan restriction enzymes of type I (2) and type II (1) were also identified with no known recognition motif. The lack of orphan methyltransferases, however, meant that DNA methylation was unlikely to be playing a role beyond defence in this MAG.

The *Paraglaciecola* MAG has two type II orphan methyltransferases, one of which recognizes GATC motif, also identified *de novo*. It also has two restriction enzymes, one type I (with no known REBASE homolog) and one type IV. Given that type IV restriction enzymes cleave methylated DNA, we can hypothesize that it plays an immune defence role. The genetic identification of an orphan methyltransferase recognizing the GATC motif, however, supports the hypothesis that *Paraglaciecola* also uses DNA methylation to regulate its DNA. With very little data in the top ice for this MAG (29 sites out of 22 094), conducting a differential methylation analysis between ice horizons for *Paraglaciecola* was not possible.

### The viral methylation landscape

By surveying methylation motifs across thousands of viral genomes, we gained new insights into the molecular strategies that may shape virus-host interactions in extreme environments. Of the 4593 viral contigs analysed, 26% (1224) did not have sufficient data for *de novo* motif identification and 70% (3257) showed no detectable methylation motif. The remaining 2% (112) of contigs together showed 36 unique motifs, the most common being **C** and **C**C. The methylation type distribution in *de novo* motifs was 70% for 5mC, 24% for 4mC, and 5% for 6 mA. By homology of genes to REBASE, we identified 29 motifs, the most common being G**A**TC, CCCGGG, and G**C**. At least one restriction-modification gene was detected on each of 359 contigs.

The genetic analysis revealed 49 viral contigs which coded for at least one restriction enzyme. Among the higher quality contigs, all but one belonged to class *Caudoviricetes,* with the remaining one belonging to *Megaviricetes. Megaviricetes* infect eukaryotes and are known to encode restriction enzymes [80, 81], whereas *Caudoviricetes*, which infect both Bacteria and Archaea, including halophilic archaea, encode complete restriction modification systems [82]. These systems are thought to have many functions: preventing superinfection, deteriorating specific mobile genetic elements, or regulating DNA replication timing [82–84].

Our viral contigs had a surplus of methyltransferases, suggesting broad presence of orphan methyltransferases in sea-ice phages (Z-score of 21.8 for Type II, equivalent to *P* < .01) (Fig. 2). Orphan methyltransferases can be advantageous to phage for many reasons, but primarily by allowing them to bypass host restriction-modification systems [84]. As viruses cannot express their own genes, methylation of the viral genome occurs via methyltransferases during infection, when the host is expressing either virally or host-coded enzymes. During viral DNA replication, methyltransferases can modify recognized motifs before the DNA is packaged into the viral capsid, where the methylated DNA persists for the lifetime of the phage [84–87]. This modification grants resistance to restriction-modification systems targeting those motifs in subsequent infection cycles.

In essence, the presence and methylation fraction of specific motifs act as a memory of phage infection history, offering clues about its passage through different hosts. This capacity to reconstruct infection histories could be particularly valuable in assessing the impacts of a dynamically stressful sea-ice environment, where hypothetically viruses could be pushed to broaden their host range [88]. By pairing motif profiles between phages and potential hosts, hypothesizing candidate infection histories becomes possible, especially in scenarios where hosts target distinct methylation motifs [89]. Such analyses demand comprehensive genomic data for both hosts and phages, including information on methyltransferase presence and motif recognition sites.

**Figure 4:**
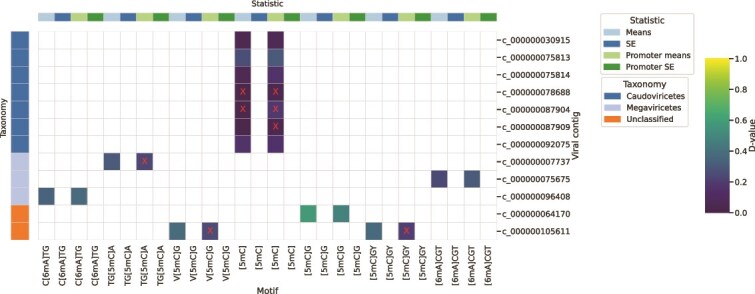
Significance of differential methylation in viral contigs between top and bottom ice. The d-value of the Kolmogorov-Smirnov similarity test is shown for each of the 12 viral contigs with sufficient data. Red “X” signifies that the statistical test result is insignificant *(P* ≥ .05), and thus the methylation distribution for this contig can be considered the same between horizons. D-value of 1 indicates largest difference in distribution, 0 indicates no difference. Many contigs show significant differences in the distribution of per-nucleotide mean methylation fraction, when considering all motif instances and only those within promoter regions. As all contigs had insufficient replicate data to calculate standard error, a population characterization cannot be made.

A comprehensive analysis of infection histories cannot be conducted with our dataset, but the data do show some evidence of different methylation states within the ice (Fig. 4). Many motifs would be expected to have similar methylation fractions across horizons, as these motifs are required for phage to bypass the RM systems of their hosts. Nevertheless, we identified eight motifs *de novo* in common to both bacteria and viruses, with evidence of differential methylation in viruses between ice horizons. Most striking is viral contig c_000000093169 (belonging to *Caudoviricetes*), with its AC**A**Y motif highly methylated in bottom ice. We identified a Type I restriction enzyme that recognizes ACAYNNNNNRTT on contig c_000000079259 and determined its taxonomy as *Polaribacter*. This taxonomic assignment is further supported by the fact that the homologous enzyme was found in *Polaribacter* sp. BM10*,* matching the results of a BLAST alignment search [90]. Given that type I enzymes are combination restriction-and-modification enzymes, this finding may suggest a recent infection of *Polaribacter* by this phage*.* No tRNA could be identified on c_000000093169 using tRNA-Scan [91] or matching CRISPR spacers on c_000000079259 using CRASS [92].

Turning to prophages, we found 39 in our dataset, most belonging to classes *Caudoviricetes* and *Prasinovirus*. Among these, five encoded restriction-modification genes, all of which were methyltransferases. Three of the methyltransferases were homologous to enzymes with known motifs: two to GATC and one to GGATG. Two MAGs (s__scgc-aaa076-p13_sp905182885__bin_0_21_12-contigs, and g__psychromonas__bin.102; Table 1) were each found to contain a prophage, neither of which coded for a methyltransferase. As the former had no restriction modification genes and no methylation motif, we did not analyse it further. Case studies of the *Psychromonas* methylome and its prophage, as well as a prophage identified in a *Pelagibacter* contig, appear below*.*

A high-quality prophage with an orphan methyltransferase was identified on contig c_000000073417 classified as *Polaribacter* sp. This contig is 45 419 bp long, including the prophage which is 36 496 bp long. The prophage was annotated as belonging to *Caudoviricetes*. An orphan type II methyltransferase was identified on this prophage as a homolog of *M.Uph13359ORFH1P* which recognizes the GATC motif. GATC was found 180 times on the prophage, and 32 times on the rest of the contig. The motif occurred 1.4× more frequently in the host than in the phage (*P* = .11). Without a complete MAG associated with this contig, we can only hypothesize the role of this methyltransferase. *Polaribacter* species are known to have multiple restriction modification systems, though none have a restriction enzyme recognizing this motif [90]. The frequent occurrence of GATC within the prophage suggests it is not under selective pressure from host restriction enzymes. Intriguingly, GATC is often linked to regulatory functions in bacteria, hinting at a possible regulatory role for this motif in both the phage and its host, potentially contributing to adaptation in the sea-ice environment. No functional annotations were available to further clarify the regulatory influence of this motif.

### Case study: *Psychromonas* methylome

The *Psychromonas* methylome reveals a dynamic interplay between prophage and host methylation patterns, offering clues about host-phage regulatory relationships in sea-ice ecosystems. A prophage belonging to *Caudoviricetes* was identified on contig c_000000087963, binned as part of our *Psychromonas* MAG. We refined the taxonomic assignment of this contig by blasting it against the NCBI core nucleotide database; the best hit was *Psychromonas ingrahamii*. A previous in-depth genomic analysis of this bacterium did not identify a prophage, though integration sites were found [93]. Of the five genomes available for *Psychromonas* on REBASE, two coded for a methyltransferase with GATC as a known recognition motif, one of which was *P. ingrahamii* [90]. This finding aligns with our *de novo* detection of a G**A**TC motif in the *Psychromonas* MAG, as well as the identification of a methyltransferase on contig c_000000028297 i.e. highly homologous to M.Pin37DamP from *P. ingrahamii.*

With the taxonomic context established, we next quantified the distribution of the GATC motif across the host and prophage genomes. In our *Psychromonas* MAG*,* the GATC motif occurs 35 280 times over, 938 485 nucleotides (excluding prophage). In the prophage, the GATC motif occurs 144 times over 32 300 nucleotides. Although no known restriction enzyme recognizing GATC was found in our MAG, restriction enzymes without a clear corresponding methyltransferase were identified. Theoretically, these enzymes could recognize the GATC motif, which would apply a selective pressure on this virus to reduce the number of GATC sites. However, given that the putative orphan methyltransferase recognizing GATC in *Psychromonas* is homologous to a Dam-like methyltransferase, methylation of the GATC motif is more likely to play a regulatory or DNA mismatch repair role [24, 72, 94, 95]. Thus, the disparity in GATC motifs, 1.6× higher in the host compared to the prophage (*P* < .0001), suggests evolutionary pressure on the phage to minimize regulatory interference. While the functional annotations of flanking genes did not provide additional insight, the absence of GATC-associated restriction enzymes coupled with the Dam-like methyltransferase homology reinforces the hypothesis that methylation here serves a purpose beyond defence.

### Case study: *Pelagibacter* methylome

**Figure 5:**
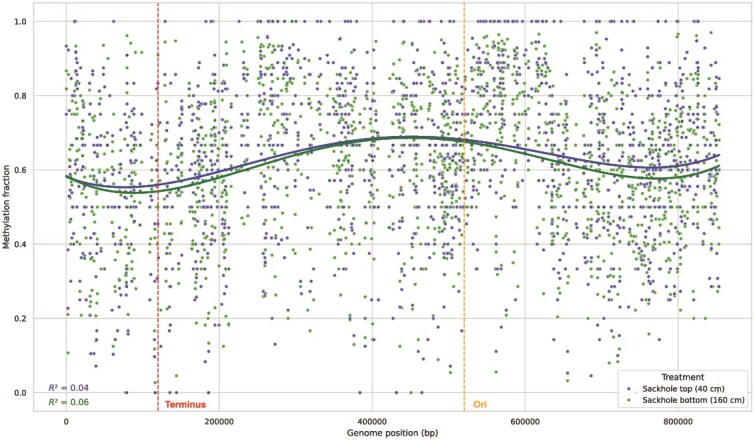
The methylome of a binned *Pelagibacter* MAG. In this MAG, the only identified methylation motif was GANTC, consistent with previous studies [21, 82]. Here we plot the methylation fraction for each instance of the 6 mA-methylated motif across the MAG for both the top and bottom ice horizons. The solid lines are a quartic fit for each treatment. The dashed vertical lines mark the terminus (red) and origin (orange) of replication. Similar variation in methylation over a genome was previously suggested to be evidence of cell cycle regulation [20].

In the hypersaline, subzero sea-ice environment, the *Pelagibacter* methylome revealed unexpected strategies for surviving extreme conditions, including methylation-mediated regulation and prophage-host interplay. We identified one *de novo* methylation motif in our *Pelagibacter* MAG: G**A**NTC. This finding is consistent with the identified methylation-related genes found in the MAG. We found one methyltransferase from a restriction modification type II system, with 99.44% homolog identity to M.PgiNP1I which has a known cognate motif of G**A**NTC previously identified in *Pelagibacter* genomes and MAGs [20, 90]. This motif has been identified consistently in other marine Alphaproteobacteria [71]. Of 1923 instances of this motif in our MAG, sufficient data for 84% (1623) were present in our dataset. The identified motif and corresponding methyltransferase were suggested previously to play a cell cycle regulator role in *Pelagibacter* [20]. This claim is supported by a variation of the mean methylation fraction across the genome between the *ori* and *ter* and based on the functioning of the *CcrM* gene in *Caulobacter crescentus*. Although we cannot definitively identify *ori* and *ter* in our MAG, the pattern of across-genome variation in methylation fraction closely resembles that reported by Seong *et al.* from the North Pacific [80] (Fig. 5). While they attributed this variation to exponential growth, the environmental conditions of sea-ice cast doubt on a relationship between the observed pattern and exponential growth occurring *in situ*, as *Pelagibacter* is not known to grow at subzero temperature and hypersalinity [96]. However, we also identified 46 motif instances that were significantly differentially methylated in *Pelagibacter* MAG between top and bottom ice (Table 2), suggesting that methylation acts as a dynamic regulatory tool in response to environmental conditions. In three instances, the motifs were within the transcription start site of a flanking gene (taken to be 60 nucleotides upstream from the start codon). The functional diversity of these genes (Table 2) suggests a broad regulatory role for DNA methylation, consistent with the identified methyltransferase acting as a cell cycle regulator. In the case of *Pelagibacter* entrained into sea-ice brine, this regulator function may be to conserve energy to survive.

**Table 2:** Annotated differentially regulated functions in the *Pelagibacter* MAG between top and bottom ice. Of the 20 motifs that were significantly differentially methylated between the top and bottom ice horizons, the functional annotations suggest that DNA methylation was not targeting a specific pathway but rather playing an overarching regulatory role on a wide array of core metabolic processes.

Methylation difference (%)^a^	Function	Annotation source
0.75	leucyl aminopeptidase [EC:3.4.11.1]	KEGG
0.67	Uncharacterized conserved protein YhdP, contains DUF3971 and AsmA2 domains (YhdR)	COG20
0.64	segregation and condensation protein A	KEGG
0.55	phosphoribosyl 1,2-cyclic phosphate phosphodiesterase [EC:3.1.4.55]	KEGG
0.40	transcription termination/antitermination protein NusA	KEGG
0.40	large subunit ribosomal protein L9	KEGG
0.40	histidinol dehydrogenase [EC:1.1.1.23]	KEGG
0.37	Anthranilate/para-aminobenzoate synthase component II (glutamine amidotransferase) (PabA) (PDB:1I1Q)	COG20
0.35	DNA polymerase III subunit beta [EC:2.7.7.7]	KEGG
0.32	imidazoleglycerol-phosphate dehydratase [EC:4.2.1.19]	KEGG
−0.26	F-type H + -transporting ATPase subunit beta [EC:7.1.2.2]	KEGG
−0.29	Fe-S cluster assembly protein SufB	KEGG
−0.29	F-type H + -transporting ATPase subunit beta [EC:7.1.2.2]	KEGG
−0.37	elongation factor Tu	KEGG
−0.39	phospholipase/carboxylesterase	KEGG
−0.39	glutamine—fructose-6-phosphate transaminase (isomerizing) [EC:2.6.1.16]	KEGG
−0.41	3-dehydroquinate dehydratase II [EC:4.2.1.10]	KEGG
−0.43	2-oxoglutarate dehydrogenase complex, dehydrogenase (E1) component, and related enzymes (SucA) (PDB:2JGD)	COG20
−0.43	small subunit ribosomal protein S11	KEGG
−0.57	cytochrome c oxidase subunit II [EC:7.1.1.9]	KEGG

a Positive values are more methylated in the bottom ice; negative values, in the top ice.

A prophage was identified on contig c_000000080048 belonging to *Pelagibacter* sp. but not the binned MAG. A BLAST search against the NCBI core nucleotide database revealed the closest match to *Pelagibacter* sp*.* UISW137, a strain isolated from under-ice seawater at our sampling site (by M Sadler of the same sampling team). Although no prophage was identified in the isolate, additional checks of our contig with VirSorter2 [97] and identification of multiple reads spanning the start of the prophage and the host genome confidently support this *Pelagibacter* prophage belonging to *Caudoviricetes*. The contig was 88 007 bp long, including the prophage which was 63 960 bp long. A type II methyltransferase was detected on the prophage which was homologous to *M.Pgl14TORF343P* which recognizes the GGATG motif. This motif was found 97 times on the prophage and 23 times in the rest of the contig. In the isolate, this motif occurs 1302 times on a genome of length, 403 101 bp. Thus, we find that this motif occurs approximately every 1045 bp in the host part of the contig, every 659 bp in the prophage, and every 1078 bp in the complete genome of the isolate and putative host. The prophage thus has a higher frequency of this motif than the contig (*P* < .0001 contig) or the isolate (*P* < .0001), suggesting a potential regulatory rather than defensive role, because selective pressure on the phage would reduce, not increase, the frequency of a motif being targeted defensively by an endonuclease. This finding aligns with the absence of cognate restriction enzymes in the *Pelagibacter* isolate, which retains only an orphan methyltransferase for G**A**TC. The methyltransferase of the prophage we detected thus likely influences gene expression by its host. The UISW 137 genome contains 34 instances of the G**A**TC motif within 70 bp of a gene transcription start site (Table S1). The function of these genes varies and includes a wide range of central metabolic functions. This range includes functions we identified as regulated by methylation in the *Pelagibacter* MAG (Table S1), which suggests that the impact of this prophage on UISW 137 would be consequential (e.g. regulation of antisigma factor). This relationship underscores the dual role of DNA methylation in sea-ice systems: a tool for viral persistence and a mediator of host adaptation under extreme conditions.

## Conclusions

Our study, in providing this characterization of DNA methylation patterns in sea-ice bacterial communities and associated viruses, has revealed that methylation serves additional functions beyond defence against foreign DNA, as traditionally mediated by restriction-modification systems [98]. Through nanopore sequencing of stepped-sackhole brines from different ice horizons, we uncovered distinct methylation signatures between the top and bottom ice, suggesting epigenetic responses to the differing environmental conditions across the ice. The methylation landscape of the sea-ice bacterial community featured 5mC methylation (97%) and many orphan methyltransferases, known for their regulatory rather than defence functions. The differential methylation patterns by ice horizon, particularly in the G**A**NTC motif in *Pelagibacter*, also point to a regulatory role for methylation, likely in response to the more extreme environmental conditions over time in the upper ice than encountered in bottom ice. Our results suggest that DNA methylation in this pelagic bacterium may facilitate survival, possibly even growth, in the challenging sea-ice environment by regulating core metabolic functions. However, further studies are needed to directly link the observed methylation patterns to a specific modulation of metabolic activity or growth.

In the viral component, we also obtained evidence of differential methylation, which we hypothesize reflects varying infection histories between ice horizons. The identification of orphan methyltransferases in sea-ice phages suggests that these phages use this known strategy to bypass host restriction-modification systems [84]. The discovery of prophages encoding methyltransferases, including in a *Pelagibacter* strain isolated from under-ice seawater at our sampling site, provides new insights into phage-host interactions in sea-ice. These prophages may influence host metabolism through methylation-based regulation, representing a previously uncharacterized mechanism of viral influence on sea-ice microbial communities similar to the influence of auxiliary metabolic genes.

Overall, our findings demonstrate that DNA methylation represents an important, previously overlooked mechanism for responding to the dynamically stressful sea-ice environment. The identification of numerous methylation motifs, many without known associated enzymes in reference databases, suggests that sea-ice communities are using unique methylation systems that warrant further investigation. Future studies with greater sequencing depth and broader sampling could further elucidate how DNA methylation contributes to microbial survival and evolution in this extreme ecosystem, potentially revealing adaptive strategies relevant to other challenging environments and extraterrestrial analogues.

## Supplementary Material

Supplemental_text_wraf198

File_S2_wraf198

File_S3_wraf198

## Data Availability

All demultiplexed reads were uploaded to NCBI SRA as part of BioProject PRJNA1227241. Assembled contigs used in this study are also published within this BioProject. This study has been conducted using E.U. Copernicus Marine Service Information; https://doi.org/10.48670/moi-00001.
