# Supplementary material for: Multiple roles of DNA methylation in sea-ice bacterial communities and associated viruses
Source: ISME J. 2025 Aug 30;19(1):wraf198. doi: 10.1093/ismejo/wraf198 (PMC12570018; doi:10.1093/ismejo/wraf198)
Supplement: Supplemental_text_wraf198 [file supplemental_text_wraf198.docx]

# Supplemental

## Sampling and Environmental History


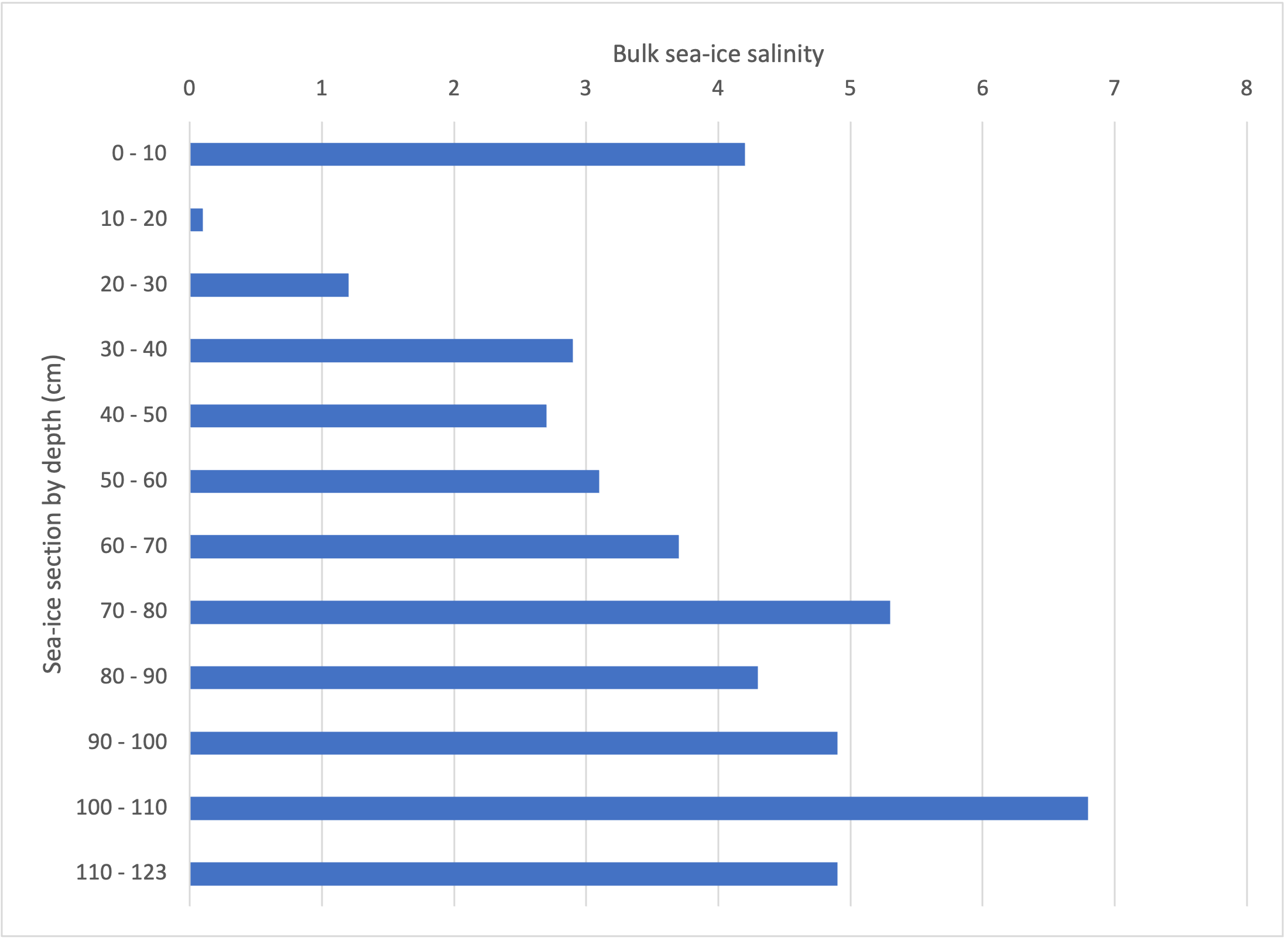


**Figure S1. Bulk salinity of a sea-ice core taken near the sackhole sampling site**. Bulk salinity was measured on 10 cm sea-ice sections, melted directly, and used to determine stepped-sackhole depths. A fresh (low salinity) section of ice at 10–20 cm is evidence of past melting, suggesting a thin patch of second year ice overlaying this predominantly first-year ice floe. No brine was possible to collect via drainage between 0 and 10 cm. Additional ice physical parameters can be found at https://doi.org/10.21334/NPOLAR.2025.44A880E0.

**Table S1.** Sackhole brine environmental parameters.

| Sample^a^ | Horizon (cm)^b^ | Brine salinity | Volume filtered (mL) | Snow cover (cm) |
| --- | --- | --- | --- | --- |
| S2-1 | 0–40 | 108 | 1750 | 11 |
| S2-2 | 40–75 | 99 | 5550 | 11 |
| S2-3 | 75–160 | 90 | 3510 | 11 |
| S3-1 | 0–40 | 105 | 620 | 2 |
| S3-2 | 40–70 | 99 | 3900 | 2 |
| S3-3 | 70–158 | 90 | 3050 | 2 |
| S4-1 | 0–40 | 96 | 150 | 5 |
| S4-2 | 40–70 | 114 | 4770 | 5 |
| S4-3 | 70–161 | N/A | 4650 | 5 |

^a^Sample notation is SX-Y, where X indicates a stepped sackhole, and Y indicates the step number; four stepped sackholes were sampled from the same sampling site, but processed samples from the first sackhole (S1) were compromised during transit to the home laboratory.

^b^0 cm is the top of the ice; total ice thickness was 209 cm.

**Table S2.** Ice-core environmental parameters and melt solution information.

| Ice core | Sample^a^ | Horizon (cm)^b^ | Freeboard (cm) | Melt solution | |
| --- | --- | --- | --- | --- | --- |
|  |  |  |  | Volume added (mL) | Salinity |
| 3 | IC3-1 | 0–30 | 18 | 1128 | 115.2–97.2 |
| 3 | IC3-4 | 30–70 | 18 | 1003 | 108 |
| 3 | IC3-2 | 70–160 | 18 | 2220 | 91.8 |
| 3 | IC3-3 | 160–207 | 18 | 1347 | 103.8 |
| 4 | IC3-1 | 0–30 | 17 | 1128 | 160.8–109.2 |
| 4 | IC3-4 | 30–70 | 17 | 1003 | 104.4 |
| 4 | IC3-2 | 70–160 | 17 | 2220 | 77.4 |
| 4 | IC3-3 | 160–205 | 17 | 1347 | 89.4 |
| 5 | IC3-1 | 0–30 | 16 | 1128 | 190.2–97.2 |
| 5 | IC3-4 | 30–70 | 16 | 1003 | 109.8 |
| 5 | IC3-2 | 70–160 | 16 | 2220 | 83.4 |
| 5 | IC3-3 | 160–205 | 16 | 1347 | 117 |

^a^Sample notation is IC3-Y, where Y indicates the horizon number; three ice-core horizons were pooled (after being melted separately) to get sufficient biomass for DNA extraction.

^b^0 cm is the top of the ice; snow cover was 8 cm.

**Figure S2. Environmental data along the floe drift track.** Over the year preceding sampling, the floe had experienced changing environmental conditions: ice thickness (upper left), ice age (upper middle), snow thickness (upper right), seawater salinity (lower left), seawater temperature (lower middle), and atmospheric temperature (lower right). Hence the bacterial community encased in the top sea-ice horizon had been subjected to larger and more extreme variations in temperature and salinity than the community near the bottom of the ice where the temperature remains near the freezing point. Note the difference in color-bar scales for seawater temperature and atmospheric temperature.

## Assembly, Metagenomics and Bin Curation

- List of contaminants removed during read quality control, NCBI identifier: 7711, 90964, 1300, 1912216, 32207, 2093 32008, 544, 579, 547, 620, 590, 1330547, 158483, 34064, 1747, and the human genome GRCh38.

**Figure S3. Read counts per sea-ice brine sample recovered from the three separate stepped sackholes (S1–S3).** Coverage was uneven between different ice horizons (top, middle, bottom); processing control yielded a minimal read count.

**Figure S4. Community composition and normalized abundance in sea-ice brine samples as determined by MEGAN6-LR**. Eukaryotes (e.g. *Polarella)* have an outsized role in community composition as our sampling strategy did not include size fractionation. *Pelagibacter* (dark orange) and *Polaribacter* (like orange) were the most abundant prokaryotes; Archaea were rare. Note that the total read count for the control was only a fraction of the sample counts (Fig. S3).

**Figure S5. Completeness (%, green) and redundancy (%, red) of the 37 curated metagenome-assembled genomes**. MAGs are identified on x-axis. We recovered 6 MAGs with high quality completeness (≥ 90%). We did not include in our methylation analyses any MAG that was either less than 30% complete or more than 10% redundant, as determined by CheckM2.

**Figure S6. Coverage of the 37 curated metagenome-assembled genomes from the sackhole brine samples and the control sample**. Note that only *Pelagibacter* (top row) had sufficient coverage in both top and bottom ice horizons for a cross-horizon comparison. MAGs with insufficient coverage in the brines were assembled thanks to the inclusion of ice-core reads in the co-assembly.

## Bacterial Methylation Landscape

**Table S3.** Functions of genes with a GGATG motif within 70 bp of their start codon in the putative UISW 137 prophage. This putative prophage may impact the regulation of these genes due to the presence of the recognized motif within or near their promoter region, thus potentially down-regulating central metabolic functions.

| **Distance from gene start** | **KEGG annotation** |
| --- | --- |
| 0 | putative tricarboxylic transport membrane protein |
| –10 | DNA repair protein RecO (recombination protein O) |
| –10 | branched-chain amino acid transport system ATP-binding protein |
| –14 | F-type H+-transporting ATPase subunit delta |
| –15 | tRNA-uridine 2-sulfurtransferase [EC:2.8.1.13] |
| –19 | single-strand DNA-binding protein |
| –25 | diaminopimelate epimerase [EC:5.1.1.7] |
| –25 | multiple antibiotic resistance protein |
| –26 | sarcosine oxidase, subunit gamma [EC:1.5.3.24 1.5.3.1] |
| –26 | sarcosine oxidase, subunit gamma [EC:1.5.3.24 1.5.3.1] |
| –26 | enoyl-[acyl-carrier protein] reductase I [EC:1.3.1.9 1.3.1.10] |
| –28 | isocitrate dehydrogenase [EC:1.1.1.42] |
| –29 | UDP-N-acetylmuramate--alanine ligase [EC:6.3.2.8] |
| –30 | methylglutamate dehydrogenase subunit B [EC:1.5.99.5] |
| –30 | sarcosine oxidase, subunit delta [EC:1.5.3.24 1.5.3.1] |
| –38 | glyceraldehyde 3-phosphate dehydrogenase (phosphorylating) [EC:1.2.1.12] |
| –38 | metalloendopeptidase OMA1, mitochondrial [EC:3.4.24.-] |
| –42 | renalase [EC:1.6.3.5] |
| –44 | ubiquinol-cytochrome c reductase cytochrome b subunit |
| –46 | uncharacterized protein |
| –49 | branched-chain amino acid transport system ATP-binding protein |
| –51 | beta-alanine--pyruvate transaminase [EC:2.6.1.18] |
| –52 | S-adenosylmethionine uptake transporter |
| –54 | protoporphyrinogen IX oxidase [EC:1.3.99.-] |
| –55 | O-antigen biosynthesis protein WbqP |
| –60 | bile acid:Na+ symporter, BASS family |
| –61 | acetylornithine deacetylase [EC:3.5.1.16] |
| –61 | enoyl-CoA hydratase [EC:4.2.1.17] |
| –64 | phosphoribosyl-ATP pyrophosphohydrolase [EC:3.6.1.31] |
| –67 | biopolymer transport protein TolR |
| –67 | maleate isomerase [EC:5.2.1.1] |
| –67 | anti-sigma B factor antagonist |
| –68 | uncharacterized protein |
| –70 | Fe-S cluster assembly protein SufD |
