# Supplementary material for: Multiple roles of DNA methylation in sea-ice bacterial communities and associated viruses
Source: ISME J. 2025 Aug 30;19(1):wraf198. doi: 10.1093/ismejo/wraf198 (PMC12570018; doi:10.1093/ismejo/wraf198)
Supplement: File_S3_wraf198 [file file_s3_wraf198.docx]

# Materials and Methods

## Ice Floe Drift

The historical drift path of the ice floe we sampled was reconstructed using sidrift commit #05075ca (Dr. Polona Itkin, <https://github.com/loniitkina/sidrift>). Environmental data were overlayed using a custom extension to sidrift. Mean daily surface atmospheric temperature was obtained from Open Meteo’s ERA5 model. Sea-ice age, sea-ice thickness, snow thickness, seawater salinity, and seawater temperature along the drift path were all obtained from the Arctic Ocean Physics Analysis and Forecast made available by Copernicus Marine from dataset ID cmems_mod_arc_phy_anfc_6km_detided_P1D-m [32, 33].

## Ice Sampling

Samples for this study were collected during the BREATHE expedition (BR7008, IMR ID #2023007008) aboard R/V *Kronsprins Haakon* in May 2023. During this expedition the ship drifted with an ice floe for 10 days, 17–27 May, over the Yermak Plateau in the Arctic Ocean north of Svalbard (Fig. 1). The sackhole brines were collected from this floe when it was located at coordinates 81° 2' 55.9464'' N, 10° 28' 41.9052'' E on 21 May.

To collect sea-ice brine, the inhabited portion of sea ice [34], from discrete horizons of the ice, we developed a stepped sackhole method. This method consists of drilling the sackhole (the void left in the ice by removing an ice core) step-by-step to pre-determined depth ranges, draining each horizon before deepening the sackhole to the next step. For each step, we allowed the brine to drain for at least 1.5 hours into the sackhole, collected the brine, and waited 30 minutes. We then checked the sackhole for brine accumulation, in which case the brine was collected, and we waited again. This wait-collect cycle was repeated until no brine drained into the sackhole, at which point we considered that horizon drained.

We selected the depth horizons to sample by determining depth ranges of roughly uniform character on the bulk salinity profile of a physical ice core sampled adjacent to our sackhole site (Fig. S1). We defined four distinct horizons: 0–10 cm, 10–40 cm, 40–70 cm, and 70–160 cm. After four hours no brine had drained into the 10 cm sackhole, so we moved to the next step and considered 0–40 cm to be the “top” (hereafter 40 cm), 40–70 cm the “middle” (70 cm), and 70–160 cm the “bottom” (160 cm). We cored four stepped sackholes, each separated by approximately 1 m, and considered them to be environmental replicates as our sampling site was generally of uniform thickness and brine salinity by ice horizon (Table S1). Adjacent stepped sackholes were not hydrologically linked on our sampling time scale (one would be empty while the other was draining), validating a separation distance of 1 m. This study is based on samples from three stepped sackholes, as those from the first one were compromised during transit to the home laboratory.

Ice was cored using a Kovacs Mark II ice coring system. Sackholes were covered with foam enclosed in a Whirl-Pak bag while waiting for drainage to occur. Brines were collected with a Masterflex field peristaltic pump into 20 L Cubitainers which were acid-washed, Milli-Q rinsed, and sample-rinsed three times prior to sample collection.

Ice core samples were obtained 2 days after the sackhole brines from the same ice floe at an adjacent site. The location of the floe at that time was 81° 2' 55.95" N 10° 28' 41.91" E. The atmospheric temperature was again –1°C. Three ice cores were taken and sectioned into the same horizons as the stepped sackholes to obtain top, middle, and bottom horizons as above. True bottom sections, from 160 cm to 205 cm (depth of the ice-ocean interface), were also collected. The core sections, pooled by horizon, were isohaline-melted, using a sterile (autoclaved) 287 ppt sodium chloride solution prepared with Milli-Q, to limit osmotic shock during melting [31]. The volume of melt solution required was determined using bulk salinity and temperature measurements [35]; the volume and salinity of added melt solution are provided in Table S2.

The physical core used to determine depth ranges for the stepped sackholes was cored on the same floe adjacent to our site on 16 May. At that time the floe was at coordinates 80° 38' 8.9664'' N and 9° 26' 8.988'' E. The core was cut into 10 cm sections which were then melted directly. The bulk salinity of each melted section was measured using a calibrated conductivity meter.

## Shipboard processing

A blank control filter was taken by filtering 100 mL of an autoclaved 287 ppt sodium chloride solution stored in acid-washed, Milli-Q rinsed Cubitainers onto a Sterivex filter prior to filtering any samples. Brines were filtered in a shipboard cold room at 1°C within 4 hours of collection, immediately upon return to the ship. They were filtered onto 0.22 μm Sterivex filters, using acid-washed, Milli-Q rinsed, autoclaved Masterflex tubing first flushed with sample. The filters were sealed using a Luer lock and Hemato-Seal, immediately flash-frozen in liquid nitrogen, and stored at –80°C until thawed for DNA extraction and sequencing. Filtrate volume was noted for each filter, and brine salinity was determined by measuring salinity of the filtrate, corrected for dilution, with a calibrated conductivity meter (Table S1). Sackhole brine samples were designated SX-Y, where X is the sackhole number, and Y is the horizon 1 for top, 2 for middle, and 3 for bottom.

## DNA Extraction and Sequencing

All samples were extracted for DNA using the Masterpure Complete DNA and RNA Purification kit following the manufacturer’s protocol for DNA purification from cell pellets with the following modifications. Sterivex filters were cracked open by applying pressure to the plastic joint with sterile pliers. Once opened, the filter was cut from its plastic structure using a sterile scalpel into two lengthwise halves. Each half was placed in a DNA LoBind tube and kept on ice. Before beginning the manufacturer’s protocol, 10 μL of lysozyme solution at a concentration of 100 mg mL^–1^ was added to the sample with 200 μL of TE buffer and allowed to digest for 30 minutes at room temperature. Additionally, the manufacturer’s protocol step for DNA pellet recovery was modified with centrifugation at 14,000 x g for 30 minutes.

Extracted DNA was quality-controlled using a NanoDrop microvolume spectrophotometer. When necessary and possible given the quantity of DNA recovered, a supplementary purification step was performed using the Zymo DNA Clean and Concentrator MagBead Kit following the manufacturer’s protocol. In later extractions, the ratio of beads to DNA was increased to 1:1 to favour DNA recovery.

Libraries for sequencing were prepared using the Oxford Nanopore Technologies Rapid Barcoding 24 V14 kit according to the manufacturer’s protocol. DNA samples were sequenced in batched runs on an Oxford Nanopore Technologies PromethION flow cell at the University of Washington’s Nanopore Sequencing Core. Sequencing output was suboptimal due to free adapter, the presence of polysaccharides, and low DNA input into the library at times. In later runs, DNA concentrations in extractions were quantified using a Qubit fluorometer which improved library quality. The last sequencing library was prepared using an Oxford Nanopore Technologies Native Barcoding 24 V14 kit. The resulting data were base-called and demultiplexed by the University of Washington’s Nanopore Sequencing Core using the SUP (superior accuracy) model and the February 2024 methylation models 5mC (all context), 6mA (all context), and 4mC (all context, research model) to obtain the highest possible base-called reads with all three methylation types detectable in any nucleotide context using Dorado (Nanopore, https://github.com/nanoporetech/dorado/).

## Assembly, Metagenomics and Bin Curation

The obtained reads were first filtered by an initial quality control step. Using Chopper v0.9.0 [36], reads shorter than 100 base pairs or with a mean Phred score below Q10 were removed. Resulting reads had an N50 length of 7,069, and average quality of Q15.25. Any reads mapping to a pre-determined set of contaminants (see Text S1) were removed. The contaminants were determined from the species present in the control sample after profiling using SingleM v0.18.3 [37]. Quality-controlled reads above 1000 base pairs were then co-assembled using Flye v2.9.5 [38] with parameters --nano-hq -meta, and any contigs shorter than 1000 bp were discarded (which removed 0.12% of total nucleotides). A co-assembly was chosen, as the inter-sample variability was expected to be low given that samples were collected at a short distance from each other on level sea ice. Moreover, due to the heterogeneous sequencing performance the number of reads obtained varied per sample; a co-assembly thus maximized the number and quality of recovered contigs. Contamination from sequencing adapters and synthetic oligos was assessed on the assembly, then removed using the NCBI FCS tool v0.5.0 [39].

To obtain gene functional annotations, genetic regions, and to curate MAGs, Anvi'o v8-dev was used [40, 41]. A contigs database was generated to which KEGG KoFams [42, NCBI COGs [43], and gene-caller annotations were added using pyrodigal v3.5.2 [44, 45]. The quality-controlled reads from each sample were then mapped onto the contigs in a profile database, and the profile databases were merged. To facilitate binning, contigs were clustered using CONCOCT v1.1.0 [46] into 10 metabins based on kmer frequency and coverage. Metabins were curated further by hand. In addition, METABAT2 v2.17 [47] was used to automatically bin contigs into MAGs.

The set of manually and automatically curated bins was reconciled manually, preferring manual bins when a contig was present in more than one bin. Automatically generated bins were also checked by blasting each contig to the NCBI RefSeq nr database and checking taxonomy for consistency within a bin. Redundancy and completeness for each MAG was assessed initially using Anvi'o and finally using CheckM2 v1.0.1 [48]. CoverM v0.7.0 was used to determine the coverage of each MAG in each sample [49]. MAGs with more than 10% redundancy were discarded.

Every MAG was searched for viral contigs and removed if present (prophages were not removed from MAGs). Viral contigs and plasmids were identified using geNomad v1.11.0 [50] and CheckV v1.0.3 [51]. Briefly, viral contigs were first identified with geNomad and then assessed using CheckV. Prophages were taken to be any subset of a contig marked by geNomad as a provirus on which at least one viral gene was present, or any contig marked as a provirus by CheckV.

To assign prokaryotic taxonomy to our MAGs we used gtdb-tk v2.3.2 [52]. Finally, to determine individual community composition, we mapped each contig onto the NCBI RefSeq nr database using Diamond [53], then used MEGAN6-LR v6.25.10 [54]. To discriminate prokaryotic and eukaryotic contigs for further analyses we used Kaiju v1.10.1 [55] , while annotations for viral and plasmid contigs were taken from geNomad and CheckV.

## Methylation Analysis

To obtain methylation data for each contig in each sample, the quality-controlled reads from each sample were mapped onto the assembly using minimap2 v2.28 [56] with parameters -ayx map-ont. Methylation data were aggregated per nucleotide using the pileup functionality in modkit v0.4.4 (Nanopore, https://github.com/nanoporetech/modkit) with a confidence threshold set to 95%. De-novo motif identification with a minimum coverage of five was done using modkit as well for each bacterial contig with sufficient data in the metagenome, for every viral contig classified by CheckV, and for every MAG. Differential methylation was assessed using modkit’s dmr pair command, which individually considered each methylation site of every instance of an identified motif in the given genetic sequence (contig or MAG) in a coverage-normalized manner. We only considered pairs with a balanced maximum a posteriori p-value of less than 0.05. Differential analysis for MAGs was conducted only when data for a given instance of a motif were available for treatments with a minimum coverage of five for the data points common to all treatments; as a result, analysis was limited by the least well-covered sample. For contig analysis, an additional criterion required both a minimum coverage threshold of 5x and ≥20% of motif instances achieving this coverage across all analysed ice horizons. This workflow was facilitated by custom scripts available at https://github.com/Ge0rges/Methylation-analysis under tag v1.0. Genes related to restriction modification systems and corresponding cognate motifs were identified using MicrobeMod v1.0.5 [57].

For each methylated nucleotide, we obtained a normalized methylation fraction as follows. For each sample, we calculated a fraction by dividing the number of reads where the nucleotide is methylated by the total number of reads. We then took a weighted mean of the per-sample fractions, weighted by each sample’s coverage, to get a methylation fraction for the nucleotide in each treatment (ice horizon). This process normalized our values by coverage and granted more weight to replicates of a treatment with deeper coverage, minimizing the impact of uneven coverage in our dataset. In the presentation of methylation data in our analysis, we defined low methylation levels to be less than 33%, medium to be between 33% and 66%, and high to be above 66%. For the *Pelagibacter* MAG whole-methylome visualization, contigs were ordered based on how they mapped to *Pelagibacter giovannoni* using MAUVE reorder [58]; the origin (*Ori*) and terminus (*ter*) were determined using gc_skew [59].

To determine significance of motif enrichment, a chi-squared test was performed on a 2x2 contingency table with the appropriate counts, and the p-value is reported for significant difference. To determine significant differences of contig methylomes between ice horizons, we used a Kolmogorov-Smirnov test and applied a Benjamini/Hochberg correction with alpha 0.01 using statsmodels v0.14.2 [60].

## Software Stack

Figures were generated with seaborn v0.13.2 [61], cartopy v0.23.0 [62], and matplotlib v3.8.4 [63]. Python v3.10.14 was used throughout. Data processing relied heavily on polars v1.9.0 [64] and pandas v2.2.2 [65]. Virtual environments were managed with conda v24.1.2 and mamba v2.0.5. Workflows used snakemake v7.32.4 [66].
